# Epidemiological pattern of COVID-19 and its association with periodontal health in an urban Indian cohort

**DOI:** 10.3389/fpubh.2023.1108465

**Published:** 2023-03-27

**Authors:** Ishita Gupta, Shivani A. Patel, Dimple Kondal, Michael Goodman, Sailesh Mohan, Mohammed K. Ali, Nikhil Tandon, K. M. Venkat Narayan, Dorairaj Prabhakaran, Krithiga Shridhar

**Affiliations:** ^1^Centre for Chronic Disease Control, New Delhi, India; ^2^Hubert Department of Global Health, Emory University, Atlanta, GA, United States; ^3^Emory Global Diabetes Research Center, Woodruff Health Sciences Center and Emory University, Atlanta, GA, United States; ^4^Department of Epidemiology, Rollins School of Public Health, Emory University, Atlanta, GA, United States; ^5^Public Health Foundation of India, Gurgaon, Haryana, India; ^6^Deakin University, Melbourne, Australia; ^7^Department of Family and Preventive Medicine, School of Medicine, Emory University, Atlanta, GA, United States; ^8^Department of Endocrinology, All India Institute of Medical Sciences, New Delhi, India; ^9^London School of Hygiene and Tropical Medicine, London, United Kingdom

**Keywords:** SARS-CoV-2, COVID-19, periodontal disease, gingival disease, oral health

## Abstract

**Background:**

Studies have highlighted a possible influence of gingival and periodontal disease (PD) on COVID-19 risk and severity. However, the evidence is based on hospital-based studies and community-level data are sparse.

**Objectives:**

We described the epidemiological pattern of SARS-CoV-2 infection in Delhi and evaluated the associations of gingival and PD with incident COVID-19 disease in a regionally representative urban Indian population.

**Methods:**

In a prospective study nested within the Centre for Cardiometabolic Risk Reduction in South-Asia (CARRS) study, participants with clinical gingival and periodontal status available at baseline (2014–16) (*n* = 1,727) were approached between October 2021 to March 2022. Information on COVID-19 incidence, testing, management, severity was collected as per the WHO case criteria along with COVID-19 vaccination status. Absolute incidence of COVID-19 disease was computed by age, sex, and oral health. Differences in rates were tested using log-rank test. Poisson regression models were used to evaluate independent associations between gingival and PD and incidence of COVID-19, adjusted for socio-demographic and behavioral factors, presence of comorbidity, and medication use.

**Results:**

Among 1,727 participants, the mean age was 44.0 years, 45.7% were men, 84.5% participants had baseline gingival or PD and 89.4% participants had received at least one dose of COVID-19 vaccine. Overall, 35% (*n* = 606) participants were tested for COVID-19 and 24% (*n* = 146/606) tested positive. As per the WHO criteria total number of cases was 210, constituting 12% of the total population. The age and sex-specific rates of COVID-19 were higher among men and older participants, but women aged >60 years had higher rates than men of same age. The incidence rate did not differ significantly between those having gingival or PD and healthy periodontium (19.1 vs. 16.5/1,000 person-years) and there was no difference in risk of COVID-19 by baseline oral disease status.

**Conclusion:**

Gingival and PD were not associated with increased risk of COVID-19.

## Introduction

COVID-19 disease caused by Severe Acute Respiratory Syndrome Coronavirus 2 (SARS-COV-2) has accounted for over 598 million cases and more than 6 million deaths across the world since first reported in 2020 (1). With more than 44 million test-confirmed cases and over five hundred thousand deaths, India is among the most affected countries worldwide (2). While most infected people have mild symptoms and recover at home, over 15% require specialized treatment such as hospitalization, intensive care, and oxygen supplementation due to severity, acute respiratory distress syndrome, and other conditions (3). Several factors influence the disease progression, which include but are not restricted to old age and comorbid conditions such as obesity, hypertension, diabetes, cardiovascular diseases (4–11).

An understudied dimension of COVID-19 risk is oral health. Studies have highlighted shared risk factors between oral and chronic diseases and COVID-19 infection and thus a possible influence of poor oral hygiene, gingival, and periodontal diseases on the risk of COVID-19 infection (12, 13) and its severity (14–19). Gingival disease (GD), characterized by poor oral hygiene and plaque biofilm harboring oral microbial pathogens deposited on dental and tissue surfaces of the oral cavity, clinically manifests as episodes of acute inflammatory changes and bleeding from gums that may be transient or persistent in nature (20). Periodontal disease (PD) is a complex, multicausal chronic inflammatory disorder leading to the destruction of soft tissues and bone surrounding the teeth (21). According to the Global Burden of Disease Study, severe PD is the 11^th^ most prevalent condition worldwide (affecting ~10.8% people) (22–24), with prevalence ranging between 30–90% in India (25).

Cross-sectional (12, 14, 16–19) and prospective (13, 15) observational studies report, over three-fold increase in severity of COVID-19 infection (and death) among persons with poor oral health or PD. However, they are limited by either sample size, lack of community level prospective data, or clinically ascertained oral health status. Therefore, we prospectively followed a regionally representative community-based population in New Delhi, a geographic region that experienced one of the highest incidence and severity of COVID-19 both in India and globally (1, 26). We utilized baseline clinical information on gingival and periodontal health to evaluate its association with incident COVID-19 and its severity.

## Materials and methods

### Study design

This was a prospective observational analysis of the Centre for Cardiometabolic Risk Reduction in South-Asia (CARRS) study. CARRS is a community-based longitudinal health assessment of 30,000 adult participants in New Delhi and Chennai, India and in Karachi, Pakistan across two urban representative cohorts initiated in 2010 and 2014, respectively (27, 28). Annual follow-ups comprise of a comprehensive questionnaire along with physical measurements and 8-h fasting blood sample collection at alternate follow-ups from men and non-pregnant women aged ≥ 20 years residing in the study areas.

### Study population and selection

From the 4,725 CARRS cohort-2 members in New Delhi, a random sub-set of 2,045 participants were enrolled in the Oral Health Study (OHS) between October 2014 and December 2016 (*N* = 2,045). They responded to the World Health Organization’s (WHO) Oral Health Assessment questionnaire and underwent detailed clinical oral examination (29). The field interviewers followed-up the OHS participants between October 2021 to March 2022 to complete telephone interviews and collect information on the history of COVID-19 and its severity. Except for those who refused, each participant was contacted five times over telephone at different times and days of the week. Participants who did not respond to phone calls were contacted by the study team through household visits to confirm loss to follow-up. Interviews were conducted by trained research staff who were blinded to the exposure status of participants.

#### Inclusion criteria

- Participants of the CARRS cohort residing in New Delhi
- Men and women (≥20 y) with baseline information on oral health status
- Participants who agreed to participate in the follow-up study with informed consent.

#### Exclusion criteria

- Men and women (≥20 y) without baseline information on oral health status
- Participants who refused to participate in the follow-up study.
- Participants who were not contacted after multiple attempts over both phone (five times) and household visit (twice) during the study.

### Ethics

The study was conducted according to the Declaration of Helsinki and was approved by Institutional Ethics Committee of the Centre for Chronic Disease Control, New Delhi (CCDC-IEC_13_2021). Informed consent was obtained from all participants before administering the questionnaire.

### Exposure assessment

Gingival and periodontal health status was ascertained at baseline by qualified and trained dentists who attended calibration workshops for assessment and interpretation of oral indices. The details are published elsewhere (29, 30) with an overview presented in Table 1.

**Table 1:** Exposure and outcome assessment.

Variable	Definition
Exposure (available from baseline assessment, 2016–18)	
Healthy	Absence of gingival/periodontal disease
Gingival disease	Measured by Community Periodontal Index modified score. Evidence of bleeding on probing, calculus, or pocket without attachment loss
Periodontal disease*	≥2 sites with pocket depth ≥ 5 mm or ≥ 2 sites with attachment loss ≥4 mm or attachment loss of ≥6 mm at 2 or more sites and a pocket depth of ≥5 mm at even one site
Outcomes** (collected at current follow-up October 2021 – March 2022)	
Incident COVID-19 cases	Includes suspect, probable or confirmed COVID-19 cases between January 2020 to March 2022
Suspect case	1. Participant meeting Clinical AND Epidemiological criteria
	Clinical
	Acute onset of fever and cough OR
	Acute onset of ANY 3 or more of the following: fever, cough, general weakness/fatigue, headache, myalgia, sore throat, coryza, dyspnea, anorexia, nausea/vomiting, diarrhea, altered mental status
	Epidemiological
	Residing or travel to area with community transmission anytime within 14 days prior to symptom onset OR
	Working in healthcare settings including within health care facilities or within community anytime within 14 days prior to symptom onset
	2. A patient with severe acute respiratory illness: acute respiratory infection with history of fever (≥ 38C) and cough, with onset within last 10 days and requires hospitalization
Probable case	1. Patient who meets clinical criteria above AND is a contact of a probable or confirmed case
	2. A person with recent onset of anosmia (loss of smell) or ageusia (loss of taste) in the absence of any other identified cause
	3. Death, not otherwise explained, in an adult with respiratory distress preceding death AND was a contact of a probable or confirmed case
	4. Physician diagnosed COVID-19 case without any test
Confirmed case	1. Positive Nucleic Acid Amplification Test (NAAT) including Reverse transcription polymerase chain reaction (RT-PCR), or any other similar test approved by Indian Council of Medical Research (ICMR)
	2. Positive SARS-CoV-2 Antigen-Rapid Diagnostic Test (RDT) AND meeting either the probable case definition or suspect criteria OR
	3. Asymptomatic person with a positive SARS-CoV-2 Antigen-RDT who is a contact of a probable or confirmed case
	4. Self-reported infection based on symptoms experienced
Severity	
Mild	Fever, cough, or fatigue AND home isolation without ventilator or oxygen support
Moderate	Difficulty in breathing or mild pneumonia AND hospitalization/home with oxygen or ventilator support
Severe	Severe pneumonia, other organ failure & possible death AND hospitalization AND oxygen or ventilator or plasma therapy COVID-19 related death

*Periodontal status has been ascertained as per Centers for Disease Control and Prevention and American Academy of Periodontology definition for population-based studies. **As per WHO’s Public Health Surveillance for COVID-19 (Interim guidance) adopted by Ministry of Health & Family welfare, India (GoI) in the Clinical Management Protocol for COVID-19.

### Outcome assessment

Outcome assessment (Table 1) was done as per the WHO COVID-19 case criteria (3, 31) based on self-reported COVID-19 history and severity. COVID-19 deaths were ascertained through verbal autopsy with next of kin (28). Participants were also asked about COVID-19 testing, care received, and vaccination status (with number of doses).

### Covariates assessment at baseline

Data on participant demographic characteristics (age, sex, monthly household income, education, and employment status), lifestyle factors (ever use of tobacco/alcohol, vigorous to moderate physical activity at work or leisure, type of diet), comorbidities (diabetes, hypertension, hyperlipidaemia, overweight/obesity, cancer, kidney disease, heart diseases, stroke), and medication history for chronic diseases were collected using interviewer-administered questionnaires. Plasma fasting blood glucose (Hexokinase), systolic and diastolic blood pressure (automated Omron HEM-7080) and anthropometric measurements (Tanita BC-418, Seca-213 Portable Stadiometer) were also available (28, 29).

### Statistical analysis

Baseline characteristics of the study participants were summarized by calculating percentages for categorical data and means and standard deviations (SD) for continuous variables. The differences in proportions and means between those reporting COVID-19 and those with no history of COVID-19 were compared using chi-square or *t*-test, respectively. Overall and stratum-specific (by age, sex, and exposure status) incidence rates of COVID-19 were calculated and differences in rates across groups was evaluated using log-rank test. Incidence rates (IR) with 95% confidence intervals (CI) were computed over the time from baseline to final available follow-up, censored for participants lost to follow-up. Person-years were estimated from the date of enrolment to the time of COVID-19 diagnosis or the last date of visit or death, whichever was documented earlier.

Multivariable Poisson regression models were used to evaluate independent associations between GD and PD and incidence of COVID-19 with results expressed as adjusted incidence rate ratios (IRR) and the corresponding 95% CI, comparing with those without GD or PD. The covariates of interest in these models included age in years (20–40/41–60/>60 age categories), sex (men/women), education status (up to secondary school/graduation and above), employment status (employed/unemployed), monthly household income in INR (≤30,000/>30,000), alcohol and tobacco consumption (yes/no), vigorous to moderate physical activity (yes/no), diet (vegetarian/non-vegetarian), presence of comorbidities mentioned above (yes/no), and any regular medication intake (yes/no). Robust standard error models were utilized to account for household clustering in our study population. The analysis was performed using STATA statistical package (StataCorp. 2011. *Stata Statistical Software: Release 12*. College Station, TX: StataCorp LP).

## Results

Among 2,045 participants with information on baseline (2016–18) gingival and periodontal health status, 84.5% (*N* = 1,727) responded to the follow-up survey from October 2021 to March 2022 (Supplementary Figure S1) either by telephone (52.7%) or during a household visit (47.3%). Demographic characteristics of respondents and non-respondents were similar (Supplementary Table S1). However, a greater proportion of respondents had graduate or above level of education (27.3% vs. 18.6%) and comorbidities (89.7% vs. 85.5%) compared to non-respondents. Respondents were also less likely to be tobacco users (21.3% vs. 31.1%,) compared to non-respondents.

Table 2 summarizes the baseline characteristics of study participants (*n* = 1,727) included in the final analysis (mean (±SD) age in years: 44.0 (±12.8); 45.7% men). Less than a third (27.3%) had graduate or above level of education, 13.7% had no formal education and more than half (55.2%) were currently unemployed. A fifth of participants were tobacco and/or alcohol users with less than a half (48.1%) doing moderate to vigorous physical activity at work or leisure and half (50.2%) were vegetarian. Nearly 90% participants had at least one comorbidity at baseline and 89.4% participants had received at least one dose of COVID-19 vaccine at the time of survey. Of the 1,727 participants who responded, 896 (51.9%) had GD, 555 (32.1%) had PD, and 276 (16%) presented with apparently healthy gingiva and periodontium at baseline.

**Table 2:** Baseline characteristics (2016–2018) of study participants (*N* = 1,727) by history of COVID-19 at follow-up (2021–2022).

Characteristics	Total		No history of COVID-19 (*N* = 1,517)		History of COVID-19* (*N* = 210)		value of *p*^@^
	*N*	%	*N*	%	*N*	%	
Socio-demographic							
Age in years**	44.0	12.8	43.3	12.3	49.1	14.9	<0.001
Sex							
Men	790	45.7	680	44.8	110	52.4	0.039
Women	937	54.3	837	55.2	100	47.6	
Educational status							
Up to secondary school	1,255	72.7	1,140	75.2	115	54.8	<0.001
Graduation and above	472	27.3	377	24.8	95	45.2	
Current employment status							
Employed	774	44.8	678	44.7	96	45.7	0.78
Un-employed***	953	55.2	839	55.3	114	54.3	
Monthly household income in INR (*N* = 1,693)							
≤30,000	1,344	77.8	1,218	80.3	126	60.0	<0.001
>30,000	349	20.2	272	17.9	77	36.7	
Lifestyle							
Consumed tobacco^	367	21.3	323	21.3	44	20.9	0.91
Consumed alcohol^	345	20.0	297	19.6	48	22.9	0.265
Did physical activity at work/recreation^^	831	48.1	759	50.0	72	34.3	<0.001
Consumed vegetarian diet	867	50.2	749	49.4	118	56.2	0.064
Co-morbidities							
Presence of at least one comorbidity^#^	1,549	89.7	1,349	88.9	200	95.3	0.005
Regular use of allopathic/modern medicine for any chronic condition	333	19.3	257	16.9	76	36.0	<0.001
COVID-19 related							
Got tested for COVID-19							
Yes	606	35.1	439	28.9	167	79.5	<0.001
No	1,121	64.9	1,078	71.1	43	20.5	
COVID-19 test results (*N* = 606)^##^							
Positive	146^$^	24.1	1	0.2	145	86.8	<0.001
Negative	460	75.9	438	99.8	22^$$^	13.2	

*Includes confirmed/probable/suspect cases as per WHO criteria. **Mean (SD). ***Unemployed includes homemaker/student/retired/un-employed. ^Ever consumed. ^^Physical Activity for at least 10 min at a time at work or leisure: “vigorous-intensity activities” require hard physical effort and cause large increases in breathing or heart rate, “moderate-intensity activities” activities require moderate physical effort and cause small increases in breathing or heart rate. #Includes hypertension (and pre-hypertension), diabetes (and pre-diabetes), hyperlipidemia, overweight/obesity, heart disease, kidney disease, stroke, and cancer. All the conditions are based on self-report by the participant following a physician-diagnosis and medication intake except hypertension, diabetes, overweight/obesity, which are also based on measured values. Hypertensive (≥140 mm Hg/90 mm Hg), pre-hypertensive (SBP: 120 mm Hg to < 140 mm Hg or DBP: 80 mm Hg to < 90 mm Hg), normotensive (<120 mm Hg/80 mmHg). Diabetes (hba1c ≥6.5 or Fasting plasma glucose (FPG) ≥126 mg/dl), Pre-diabetes (hba1c ≥5.7 to < 6.5) or (FPG 100 mg/dl to < 126 mg/dl) and Normoglycemic (hba1c < 5.7 and FPG < 100 mg/dl). Overweight/obesity BMI ≥23 kg/m^2^. ##Includes only those who reported getting a test done. ^$^One participant among those who tested positive did not report any further COVID-19 related information and was considered missing. ^$$^Twenty-two participants met WHO case criteria despite a negative test. Thus cases (*n* = 210) comprised of those tested positive (*n* = 145) and those identified based on WHO criteria despite negative test result (*n* = 22) and not getting tested (*n* = 43). @value of *p* for differences in means/median/proportions were from *t*-test, Wilcoxon Rank test or Chi-Square test, respectively.

A total of 606 participants (35.1%) reported getting tested for COVID-19, and of those 146 (24.1%) tested positive (out of which one participant was considered missing due to missing other relevant COVID-19 information). We classified our study participants for COVID-19 as per the WHO case criteria (Table 1): 210 (12.2%) participants with COVID-19 included 169 (80.5%) confirmed cases, 33 (15.7%) suspected cases and 8 (3.8%) probable cases. Among COVID-19 cases, one episode (77.6%) and mild infection (82.9%) were common wherein about 70% experienced three or more symptoms and less than a 10% required additional support in terms of oxygen/assisted ventilation. Comparison of baseline characteristics (Table 2), by differences in means, revealed that participants with COVID-19 were older in age compared to those who did not have COVID-19 (mean (±SD) 49.1 (±14.9) vs. 43.3 (±12.3) years). By differences in proportion, men (52.4% vs. 47.6%) with lower education status (54.8% vs. 45.2%), household income (60% vs. 36.7%) and comorbidities (95.3% vs. 4.7%) were more likely to be cases compared to their counterparts.

The overall incidence rate of COVID-19 was 18.64/1,000 person-years (95% CI: 16.3–21.3/1,000 person-years) (Table 3). Men reported greater overall incidence rates compared to women (21.4; 95% CI: 17.8–25.9 vs. 16.3; 95% CI: 13.4–19.8/1,000 person-years, *p*: 0.034). The incidence rates increased significantly with age [20–40 years: 13.6 (95% CI: 10.7–17.2)], 41–60 years: 18.6 [95% CI: 15.1–22.8), >60 years: 38.1 (95% CI: 28.9–50.3)/1,000 person-years, *p* < 0.001]. A similar pattern was observed when incidence rates were estimated for confirmed cases only (*N* = 169). Men of age-group 20–40 years had higher incidence rates compared to women that narrowed in 40–60 years and disappeared in the >60 years group 38.4 [(95% CI: 24.8–59.5)/1,000 person-years in women vs. 37.9 (95% CI: 26.5–54.2)/1,000 person-years in men]. However, among confirmed cases, men reported higher incidence rates than women in the same age group throughout. Additionally, the incidence rates did not differ significantly between those having GD/PD and healthy periodontium [19.1 (95% CI:16.5–22.1) vs. 16.5 (95% CI:11.5–23.6)/1,000 person-years, *p*: 0.394)].

**Table 3:** COVID-19 incidence rates (per 1,000) by age, gender, and exposure status (*N* = 1,727).

Incidence rates	Person-year	With confirmed, probable, and suspect (*N* = 210) cases			With confirmed (*N* = 169) cases only^		
		Event	Rate* (95% CI)	*p* value	Event	Rate* (95% CI)	*p* value**
Overall	11,266.2	210	18.6 (16.3–21.3)		169	15.0 (12.9–17.4)	
By age-group							
20–40 y	5,000.3	68	13.6 (10.7–17.2)	<0.001	58	11.6 (9.0–15.0)	0.001
41–60 y	4,953.1	92	18.6 (15.1–22.8)		79	15.9 (12.8–19.9)	
>60 y	1,312.8	50	38.1 (28.9–50.3)		32	24.4 (17.2–34.5)	
By age-group among men							
Overall	5,131.6	110	21.4 (17.8–25.9)	<0.001	89	17.3 (14.1–21.3)	0.001
20–40 y	2,163.6	38	17.6 (12.8–24.1)		33	15.2 (10.8–21.4)	
41–60 y	2,176.6	42	19.3 (14.3–26.1)		36	16.5 (11.9–22.9)	
>60 y	791.4	30	37.9 (26.5–54.2)		20	25.3 (16.3–39.2)	
By age-group among women							
Overall	6,134.5	100	16.3 (13.4–19.8)	<0.001	80	13.0 (10.5–16.2)	0.001
20–40 y	2,836.7	30	10.6 (7.4–15.13)		25	8.8 (6.0–13.0)	
41–60 y	2,776.5	50	18.0 (13.7–23.8)		43	15.5 (11.5–20.9)	
>60 y	521.3	20	38.4 (24.8–59.5)		12	23.0 (13.0–40.5)	
Incidence rates by exposure status							
Gingival/periodontal disease	9,447.4	180	19.1 (16.5–22.1)	0.394	140	14.8 (12.6–17.5)	0.813
Healthy periodontium	1,818.8	30	16.5 (11.5–23.6)		29	15.9 (11.1–22.9)	

^Confirmed cases as per WHO criteria. *Incidence rate per 1,000. **Log-rank test. *p*-value for overall sex specific incidence rate between men and women are 0.034 for all cases and 0.052 for confirmed cases only.

Unadjusted and adjusted IRR (95% CI) for the association between baseline oral disease status of study participants and COVID-19 incidence is presented in Table 4. The crude IRR (95% CI) of risk of COVID-19 was 1.16 (95% CI: 0.80–1.67) among those with baseline oral disease compared to those without any oral disease. On adjusting for all known important confounders and/or competing exposures with inverse probability weighting, no association was found between baseline oral disease and incidence of COVID-19 among the study participants (IRR: 1.05, 95% CI: 0.72–1.52). The findings remained unchanged when analyzed for GD and PD separately or confirmed cases only. Due to small outcome numbers, we limited our explorations to bivariate differences in proportions of GD and PD with COVID-19 severity and did not find any significant difference (Supplementary Figure S2).

**Table 4:** Unadjusted and adjusted incidence rate ratio for the association of COVID-19 incidence with baseline oral disease of study participants aged ≥20 years.

Exposure	Incidence rate ratio (95% confidence interval)					
	Crude/unadjusted (*N* = 1,727)	Age-sex adjusted (*N* = 1,727)	Model-1* (*N* = 1,727)	Weighted model-1*^ (*N* = 1,727)	Model-2^#^ (*N* = 1,683)	Weighted model-2^#^^ (*N* = 1,683)
All COVID-19 cases (*N* = 210)						
No oral disease	Reference	Reference	Reference	Reference	Reference	Reference
Any oral disease^+^	1.16 (0.80–1.67)	0.94 (0.65–1.37)	1.06 (0.73–1.54)	1.05 (0.72–1.52)	1.0 (0.67–1.48)	0.98 (0.66–1.45)
Gingival disease	1.23 (0.84–1.81)	1.10 (0.75–1.61)	1.16 (0.80–1.69)	1.14 (0.79–1.67)	1.09 (0.73–1.62)	1.07 (0.72–1.59)
Periodontal disease	1.04 (0.68–1.81)	0.65 (0.42–1.01)	0.81 (0.52–1.28)	0.80 (0.51–1.26)	0.74 (0.44–1.24)	0.72 (0.43–1.20)
COVID-19 confirmed cases^++^ only (*N* = 169)						
Exposure	Crude/unadjusted (*N* = 1,727)	Age-sex adjusted (*N* = 1,727)	Model-1* (*N* = 1,727)	Weighted Model-1*^ (*N* = 1,727)	Model-2^#^ (*N* = 1,683)	Weighted model-2^#^^ (*N* = 1,683)
No oral disease	Reference	Reference	Reference	Reference	Reference	Reference
Any oral disease^+^	0.93 (0.63–1.36)	0.80 (0.54–1.18)	0.96 (0.65–1.42)	0.93 (0.63–1.38)	0.97 (0.65–1.46)	0.95 (0.63–1.42)
Gingival disease	1.09 (0.73–1.62)	0.98 (0.66–1.46)	1.08 (0.73–1.60)	1.06 (0.71–1.56)	1.08 (0.72–1.63)	1.06 (0.71–1.60)
Periodontal disease	0.68 (0.43–1.08)	0.46 (0.28–0.75)	0.62 (0.37–1.04)	0.64 (0.36–1.01)	0.66 (0.39–1.13)	0.64 (0.38–1.09)

*Adjusted for age, sex, education status, employment status, monthly household income, alcohol and tobacco consumption, physical activity, diet, comorbidity, any regular medication intake. #Adjusted for age, sex, education status, employment status, monthly household income, alcohol and tobacco consumption, physical activity, diet, comorbidity, any regular medication intake, time to last dental visit at current follow-up. ^Poisson-regression with inverse probability weighting to account for loss to follow-up and missing co-variates and with robust standard error to account for household clusters. +gingival/periodontal disease. ++ Confirmed cases identified through test results and self-report.

## Discussion

In this study of a representative urban Indian population in New Delhi, diagnosed COVID-19 disease (as per WHO COVID-19 case criteria based on self-reported COVID-19 history and severity) was observed among 12% of participants with an overall incidence rate of 18.6/1,000 person-years. The overall incidence rate was higher among men than women and age-specific incidence rates were significantly higher with older age among both men and women. However, our study findings do not support the hypothesis that poor oral health is associated with increased risk of COVID-19.

The proportion of new cases observed in a sub-population of CARRS is similar to that reported by Larvin et al., (13) from participants across United Kingdom, another country with a high cumulative case load (23,840,513) and COVID-19 deaths (2,07,375) comparable to India (1). Although the COVID-19 cases reported in our study included those assessed using the WHO’s clinical and epidemiological criteria, among those tested for COVID-19, an overall 24% test positivity was observed which also corresponds to a high-test positivity rate (> 20%) as reported in several regions of India including New Delhi in April, 2021 during the peak of second wave (32).

The incidence rates observed in our study are consistent with those reported in other countries and settings (6, 10, 11, 33). It is noteworthy that the differences in incidence rates among men and women narrowed with advancing age and older women (>60 years) tended to have higher incidence rates than men of the same age; an observation previously reported elsewhere (34). Though early epidemiological studies reported lower risk of COVID-19 in women (33, 35), the gender differences reduced with the subsequent waves of the pandemic (36, 37).

Our finding on the association between baseline oral disease and risk of COVID-19 was in disagreement with the results of an earlier study by Anand and colleagues (in India) who reported significantly increased risk of COVID-19 infection with gingivitis, and severe periodontitis (12). This could be due to the differences in study design (case–control vs. prospective) and settings (hospital-based vs. community). On balance, the existing literature suggests that while severity of COVID-19 infection may be positively associated with oral health indicators including PD (14–19), the occurrence of COVID-19 may be unrelated to oral health (13). One of the largest available studies, examined retrospective UK Biobank data on oral health status from 13,253 adults and also reported no association between poor oral health (loose teeth or painful, bleeding gums) and risk of COVID-19 infection. Nevertheless, the observed results might have also been affected by systematic error, such as uncontrolled confounding or insufficient statistical power to detect precise associations due to the high prevalence of GD and PD at baseline in our study population and shared yet competing risk factors such as age and comorbid conditions. Due to the small sample size, we were limited in our ability to test the association between baseline oral disease and severity of COVID-19, however, crude analysis revealed no significant difference. The UK study reported a 70% increased risk of death due to COVID-19 among participants with painful or bleeding gums (indicating the severity) compared to the control group (13). It also worth noting that the association with “bleeding or painful gums” may be different from the association with “loose teeth”; however, a more in-depth understanding of this difference will require further evaluations.

Other studies focusing on COVID-19 severity or complications such as assisted ventilation, intensive care admission and deaths report 3 to 36 times higher odds of any COVID-19 complication among periodontally compromised individuals (14–16, 18, 19, 38) compared to those with healthy periodontium. Despite reporting statistically significant results, the wide confidence intervals indicate low precision due to small sample size. Other limitations of these studies include convenience sampling, relative paucity of prospective data, lack of data from clinical oral examinations, and limited information on recent access to oral care (Supplementary Table S2, 12–19, 39–43). Additionally, only one of the previous studies had a representative population-based sample (13) and sample for most studies comprised of COVID-19 positive cases to explore the association with severity rather than risk (14–19).

A plausible biological explanation for the association of GD and PD with COVID-19 risk and severity is the increase in the expression of viral adhesion receptors such as ACE2 on the mucosa of oral cavity, galectin-3 (44–48) and transmembrane protease serin 2 (TMPRSS2), on oral epithelium which is necessary for activation of the SARS-CoV-2 S-protein to bind with host cells and increase its infectivity (49, 50). Additionally, oral bacterial dysbiosis, defined as shift from healthy microbiome toward pathogenic gram-negative anaerobic genera (51), associated with PD may lead to bacterial co-infection through aspirated gingival crevicular fluid thereby initiating inflammatory changes including “cytokine storm” (52, 53).

Ours is one of the largest population-based studies with a prospective design from a low- and middle-income setting to quantify the association between GD/PD and COVID-19 risk in one of the worst affected regions. New Delhi reported 2,002,772 cases with over 26,500 deaths (26) as on September 2022. Additional strengths of our study include the objective clinical assessment data on oral health status (for exposure) and a comprehensive questionnaire administered directly to the participants or household members (for deceased participants) to gather COVID-19 information (outcomes). Use of the WHO COVID-19 case criteria based on self-reported COVID-19 history and severity (31) addresses the concerns of low testing rates during the peak waves of the pandemic (6).

An important limitation of our study was the inability to consider the time-dependent nature of the main exposure, i.e., changes in GD or PD status during follow-up. We attempted to partly account for this by adjusting for access to oral care during this time-period. Although, this could have missed those who newly developed oral disease during the recent period and did not access care, this was unlikely to have influenced our findings as a small proportion of participants presented with apparently healthy gingiva and periodontium at baseline. As the outcome was self-reported, there was a likelihood of recall bias in reporting details of COVID-19 infection, symptoms, and management. However, respondents tend to remember such uncommon/severe events in recent past. Another limitation due to the prospective nature of the study is the loss to follow-up, especially given the migration of people during the pandemic. However, the loss to follow-up was just over 15% and baseline characteristics of respondents with those lost to follow-up did not differ largely.

In summary, although we did not find an association between poor gingival/periodontal health and increased risk of COVID-19, our study provides insights into the epidemiological pattern of COVID-19 at a community level. Studies, such as ours provide an opportunity to explore novel links between oral health and systemic conditions enabling a better understanding of the disease pathways and strategies to manage them (54) and to prospectively follow participants and assess immediate and delayed complications (55).

## Data availability statement

The datasets presented in this article are not readily available because of privacy issues. Requests to access the datasets should be directed to the Indian principal investigator of the CARRS study (DP at dprabhakaran@ccdcindia.org).

## Ethics statement

The studies involving human participants were reviewed and approved by Institutional Ethics Committee-Centre for Chronic Disease Control, New Delhi, India. The ethics committee waived the requirement of written informed consent for participation.

## Author contributions

IG and KS contributed to conceptualization, project administration, data curation, analysis, interpretation, writing, and reviewing the manuscript. DK did the formal analysis and interpretation, reviewing and editing of manuscript. SP, MG, SM, MA, NT, KN, and DP contributed to conceptualization and reviewing and editing of manuscript. All authors contributed to the article and approved the submitted version.

## Funding

The work was supported by the Cardiometabolic Risk Reduction in South Asia Surveillance Study was funded in part by the National Heart, Lung, and Blood Institute (NHLBI) of the National Institutes of Health (NIH), Department of Health and Human Services [Contract no. HHSN268200900026C] and the United Health Group (Minneapolis, MN, USA). Research reported in this publication is a sub-study of the CARRS study supported by the National Heart, Lung, And Blood Institute of the National Institutes of Health under Award Number P01HL154996. Several members of the research team were/are supported by the Fogarty International Center (FIC) and the Eunice Kennedy Shriver National Institute of Child Health & Human Development, NIH through Grant Number 1 D43 HD065249, FIC at the NIH under Award Number D43TW009135 and NIH, National Cancer Institute grant number P20CA210298-01. The content is solely the responsibility of the authors and does not necessarily represent the official views of the National Institutes of Health.

## Conflict of interest

The authors declare that the research was conducted in the absence of any commercial or financial relationships that could be construed as a potential conflict of interest.

## Publisher’s note

All claims expressed in this article are solely those of the authors and do not necessarily represent those of their affiliated organizations, or those of the publisher, the editors and the reviewers. Any product that may be evaluated in this article, or claim that may be made by its manufacturer, is not guaranteed or endorsed by the publisher.

## Abbreviations

CARRS: Centre for Cardiometabolic Risk Reduction in South-Asia Study
GD: Gingival Disease
IRR: Incidence Rate Ratio
OHS: Oral Health Study
PD: Periodontal Disease
SARS-COV-2: Severe Acute Respiratory Syndrome Coronavirus-2
SD: Standard Deviation
WHO: World Health Organization

## References

[1] Johns Hopkins University and Medicine Coronovirus Resource Centre. (2021). COVID-19 Dashboard. Available at: https://coronavirus.jhu.edu/map.html (Accessed September 30, 2022)

[2] World Health Organisation. (2021). WHO COVID-19 dashboard. Available at: https://covid19.who.int/ (Accessed September 30, 2022)

[3] Government of India. (2022). Ministry of Health and Family Welfare. Available at: https://www.mohfw.gov.in/ (Accessed October 1, 2022)

[4] Holt H Talaei M Greenig M Zenner D Symons J Relton C. Risk factors for developing COVID-19: a population-based longitudinal study (COVIDENCE UK). Thorax. (2022) 77:900–12. doi: 10.1136/thoraxjnl-2021-217487 34848555

[5] Pfützner A Lazzara M Jantz J. Why do people with diabetes have a high risk for severe COVID-19 disease?-a dental hypothesis and possible prevention strategy. J Diabetes Sci Technol. (2020) 14:769–71. doi: 10.1177/1932296820930287 32506937 PMC7673189

[6] Laxminarayan R Chandra Mohan B Vinay TG Arjun Kumar KV Wahl B Lewnard JA. SARS-CoV-2 infection and mortality during the first epidemic wave in Madurai, South India: a prospective, active surveillance study. Lancet Infect Dis. (2021) 21:1665–76. doi: 10.1016/S1473-3099(21)00393-5 34399090 PMC8363227

[7] Barman Roy D Gupta V Tomar S Gupta G Biswas A Ranjan P. Epidemiology and risk factors of COVID-19-related mortality. Cureus. (2021) 13:e20072. doi: 10.7759/cureus.20072 34987936 PMC8719433

[8] Centers fror Disease Control and Prevention (CDC). COVID-19. Available at: https://www.cdc.gov/coronavirus/2019-ncov/need-extra-precautions/people-with-medical-conditions.html (Accessed October 1, 2022)

[9] Grasselli G Zangrillo A Zanella A Antonelli M Cabrini L Castelli A. Baseline characteristics and outcomes of 1591 patients infected with SARS-CoV-2 admitted to ICUs of the Lombardy region, Italy. JAMA. (2020) 323:1574. doi: 10.1001/jama.2020.5394 32250385 PMC7136855

[10] Zhang X Tan Y Ling Y Lu G Liu F Yi Z. Viral and host factors related to the clinical outcome of COVID-19. Nature. (2020) 583:437–40. doi: 10.1038/s41586-020-2355-0, PMID: 32434211

[11] Gallo Marin B Aghagoli G Lavine K Yang L Siff EJ Chiang SS. Predictors of COVID-19 severity: a literature review. Rev Med Virol. (2021) 31:1–10. doi: 10.1002/rmv.2146 PMC7855377 32845042

[12] Anand PS Jadhav P Kamath KP Kumar SR Vijayalaxmi S Anil S. A case-control study on the association between periodontitis and coronavirus disease (COVID-19). J Periodontol. (2022) 93:584–90. doi: 10.1002/JPER.21-0272, PMID: 34347879

[13] Larvin H Wilmott S Wu J Kang J. The impact of periodontal disease on hospital admission and mortality during COVID-19 pandemic. Front Med. (2020) 7:1–7. doi: 10.3389/fmed.2020.604980 PMC7719810 33330570

[14] Alnomay N Alolayan L Aljohani R Almashouf R Alharbi G. Association between periodontitis and COVID-19 severity in a tertiary hospital: a retrospective cohort study. Saudi Dent J. (2022) 34:623–8. doi: 10.1016/j.sdentj.2022.07.001 35915835 PMC9327183

[15] Costa CA Vilela ACS Oliveira SA Gomes TD Andrade AAC Leles CR. Poor oral health status and adverse COVID-19 outcomes: a preliminary study in hospitalized patients. J Periodontol. (2022) 93:1889–901. doi: 10.1002/JPER.21-0624 35294780 PMC9088593

[16] Mishra S Gupta V Rahman W Gazala MP Anil S. Association between periodontitis and COVID-19 based on severity scores of HRCT chest scans. Dent J. (2022) 10:106. doi: 10.3390/dj10060106 PMC9222103 35735648

[17] Gupta S Mohindra R Singla M Khera S Sahni V Kanta P. The clinical association between periodontitis and COVID-19. Clin Oral Invest. (2022) 26:1361–74. doi: 10.1007/s00784-021-04111-3 PMC8390180 34448073

[18] Said KN Al-Momani AM Almaseeh JA Marouf N Shatta A Al-Abdulla J. Association of periodontal therapy, with inflammatory biomarkers and complications in COVID-19 patients: a case control study. Clin Oral Investig. (2022) 26:6721–32. doi: 10.1007/s00784-022-04631-6 PMC9643194 35906340

[19] Marouf N Cai W Said KN Daas H Diab H Chinta VR. Association between periodontitis and severity of COVID-19 infection: a case–control study. J Clin Periodontol. (2021) 48:483–91. doi: 10.1111/jcpe.13435 33527378 PMC8014679

[20] Page RC. Gingivitis*. J Clin Periodontol. (1986) 13:345–55. doi: 10.1111/j.1600-051X.1986.tb01471.x 3522644

[21] Loos BG Van Dyke TE. The role of inflammation and genetics in periodontal disease. Periodontol 2000. (2020) 83:26–39. doi: 10.1111/prd.12297 32385877 PMC7319430

[22] Frencken JE Sharma P Stenhouse L Green D Laverty DDT. Global epidemiology of dentalcaries and severe periodontitis–a comprehensive review. J Clin Periodontol. (2017) 44:S94–S105. doi: 10.1111/jcpe.12677, PMID: 28266116

[23] Vos T Abajobir AA Abate KH Abbafati C Abbas KM Abd-Allah F. Global, regional, and national incidence, prevalence, and years lived with disability for 328 diseases and injuries for 195 countries, 1990–2016: a systematic analysis for the global burden of disease study 2016. Lancet. (2017 Sep) 390:1211–59. doi: 10.1016/S0140-6736(17)32154-2, PMID: 28919117 PMC5605509

[24] Bernabe E Marcenes W Hernandez CR Bailey J Abreu LG Alipour V. Global, regional, and National Levels and trends in burden of Oral conditions from 1990 to 2017: a systematic analysis for the global burden of disease 2017 study. J Dent Res. (2020) 99:362–73. doi: 10.1177/0022034520908533, PMID: 32122215 PMC7088322

[25] Shewale AH Gattani DR Bhatia N Mahajan R Saravanan SP. Prevalence of periodontal disease in the general population of India-a systematic review. J Clin Diagn Res. (2016) 10:ZE04–9. doi: 10.7860/JCDR/2016/17958.7962 PMC4963791 27504431

[26] Government of Delhi Department of Information & Publicity. Delhi fights Corona. Available at: https://delhifightscorona.in/ (Accessed September 30, 2022)

[27] Nair M Ali MK Ajay VS Shivashankar R Mohan V Pradeepa R. CARRS surveillance study: design and methods to assess burdens from multiple perspectives. BMC Public Health. (2012) 12:701. doi: 10.1186/1471-2458-12-701 22928740 PMC3491014

[28] Kondal D Patel SA Ali MK Mohan D Rautela G Gujral UP. Cohort profile: the center for cArdiometabolic risk reduction in South Asia (CARRS). Int J Epidemiol. (2022) 51:e358–71. doi: 10.1093/ije/dyac014 35138386 PMC9749725

[29] Rawal I Ghosh S Hameed SS Shivashankar R Ajay VS Patel SA. Association between poor oral health and diabetes among Indian adult population: potential for integration with NCDs. BMC Oral Health. (2019) 19:191. doi: 10.1186/s12903-019-0884-4 31429749 PMC6701092

[30] Page RC Eke PI. Case definitions for use in population-based surveillance of periodontitis. J Periodontol. (2007) 78:1387–99. doi: 10.1902/jop.2007.060264 17608611

[31] WHO. (2022). Public health surveillance for COVID-19. Available at: https://www.who.int/publications/i/item/who-2019-nCoV-surveillanceguidance-2020.8

[32] Ritchie Hannah Mathieu Edouard Rodés-Guirao Lucas Appel Cameron Giattino Charlie Ortiz-Ospina Esteban. (2020). Coronavirus pandemic (COVID-19). Available at: https://ourworldindata.org/coronavirus (Accessed October 3, 2022)

[33] Chen N Zhou M Dong X Qu J Gong F Han Y. Epidemiological and clinical characteristics of 99 cases of 2019 novel coronavirus pneumonia in Wuhan, China: a descriptive study. Lancet (London, England). (2020) 395:507–13. doi: 10.1016/S0140-6736(20)30211-7 32007143 PMC7135076

[34] O’Brien J Du KY Peng C. Incidence, clinical features, and outcomes of COVID-19 in Canada: impact of sex and age. J Ovarian Res. (2020) 13:137. doi: 10.1186/s13048-020-00734-4 33234144 PMC7684854

[35] Mazumder A Arora M Bharadiya V Berry P Agarwal M Behera P. SARS-CoV-2 epidemic in India: epidemiological features and in silico analysis of the effect of interventions. F1000Res. (2020) 9:315. doi: 10.12688/f1000research.23496.2 32528664 PMC7262570

[36] Global Health. (2020). 50/50. Available at: https://globalhealth5050.org/covid19/age-and-sex-data/ (Accessed 2022 September 29)

[37] World Health Organization. (2020). World Health Organization. *Coronavirus disease (COVID-19) situation report–198*.

[38] Shamsoddin E. Is periodontitis associated with the severity of COVID-19? Evid Based Dent. (2021) 22:66–8. doi: 10.1038/s41432-021-0179-x 34172910 PMC8226334

[39] Kamel AHM Basuoni A Salem ZA Abubakr N. The impact of oral health status on COVID-19 severity, recovery period and C-reactive protein values. Br Dent J. (2021) 1–7. doi: 10.1038/s41415-021-2656-1 PMC7904030 33627848

[40] Gardelis P Zekeridou A Suh N Le Terrier C Stavropoulos A Giannopoulou C. A pilot clinical and radiographic study on the association between periodontitis and serious COVID-19 infection. Clin Exp Dent Res. (2022) 8:1021–7. doi: 10.1002/cre2.610 35932180 PMC9562574

[41] Kaur A Sandhu HS Sarwal A Bhagat S Dodwad R Singh G. Assessment of correlation of COVID-19 infection and periodontitis- a comparative study. J Fam Med Prim care. (2022) 11:1913–7. doi: 10.4103/jfmpc.jfmpc_1978_21 PMC9254790 35800529

[42] Katz J Yue S Xue W. Dental diseases are associated with increased odds ratio for coronavirus disease 19. Oral Dis. (2022) 28:991–3. doi: 10.1111/odi.13653 32989904 PMC8004536

[43] Wang Y Deng H Pan Y Jin L Hu R Lu Y. Periodontal disease increases the host susceptibility to COVID-19 and its severity: a Mendelian randomization study. J Transl Med. (2021) 19:528. doi: 10.1186/s12967-021-03198-2 34952598 PMC8708510

[44] Badran Z Gaudin A Struillou X Amador G Soueidan A. Periodontal pockets: a potential reservoir for SARS-CoV-2? Med Hypotheses. (2020) 143:109907. doi: 10.1016/j.mehy.2020.109907 32504927 PMC7833827

[45] Xu H Zhong L Deng J Peng J Dan H Zeng X. High expression of ACE2 receptor of 2019-nCoV on the epithelial cells of oral mucosa. Int J Oral Sci. (2020) 12:8. doi: 10.1038/s41368-020-0074-x 32094336 PMC7039956

[46] Takahashi Y Watanabe N Kamio N Kobayashi R Iinuma T Imai K. Aspiration of periodontopathic bacteria due to poor oral hygiene potentially contributes to the aggravation of COVID-19. J Oral Sci. (2020) 63:1–3. doi: 10.2334/josnusd.20-0388 33177276

[47] Bertolini M Pita A Koo S Cardenas A Meethil A. Periodontal disease in the COVID-19 era: potential reservoir and increased risk for SARS-CoV-2. Pesqui Bras Odontopediatria Clin Integr. (2020) 20:162. doi: 10.1590/pboci.2020.162

[48] Kara C Çelen K Dede FÖ Gökmenoğlu C Kara NB. Is periodontal disease a risk factor for developing severe Covid-19 infection? The potential role of Galectin-3. Exp Biol Med (Maywood). (2020) 245:1425–7. doi: 10.1177/1535370220953771 32838557 PMC7553094

[49] Sakaguchi W Kubota N Shimizu T Saruta J Fuchida S Kawata A. Existence of SARS-CoV-2 entry molecules in the Oral cavity. Int J Mol Sci. (2020) 21:6000. doi: 10.3390/ijms21176000 32825469 PMC7503451

[50] Hoffmann M Kleine-Weber H Schroeder S Krüger N Herrler T Erichsen S. SARS-CoV-2 cell entry depends on ACE2 and TMPRSS2 and is blocked by a clinically proven protease inhibitor. Cell. (2020) 181:e8:271–80. doi: 10.1016/j.cell.2020.02.052 PMC7102627 32142651

[51] Kilian M Chapple ILC Hannig M Marsh PD Meuric V Pedersen AML. The oral microbiome – an update for oral healthcare professionals. Br Dent J. (2016) 221:657–66. doi: 10.1038/sj.bdj.2016.865, PMID: 27857087

[52] Hayata M Watanabe N Tamura M Kamio N Tanaka H Nodomi K. The Periodontopathic bacterium fusobacterium nucleatum induced Proinflammatory cytokine production by human respiratory epithelial cell lines and in the lower respiratory organs in mice. Cell Physiol Biochem. (2019) 53:49–61. doi: 10.33594/000000120 31169991

[53] Sahni V Gupta S. COVID-19 & periodontitis: the cytokine connection. Med Hypotheses. (2020) 144:109908. doi: 10.1016/j.mehy.2020.109908 32534336 PMC7832148

[54] Khanji MY Aung N Chahal CAA Petersen SE. COVID-19 and the UK biobank-opportunities and challenges for research and collaboration with other large population studies. Front Cardiovasc Med. (2020) 7:156. doi: 10.3389/fcvm.2020.00156 33195444 PMC7481380

[55] World Health Organization. (2021). A clinical case definition of post COVID-19 condition by a Delphi consensus. Geneva. 1–27. Available at: https://www.who.int/publications/i/item/WHO-2019-nCoV-Post_COVID-19_condition-Clinical_case_definition-2021.1 (Accessed October 1, 2022).

