# Supplementary material for: Epidemiological pattern of COVID-19 and its association with periodontal health in an urban Indian cohort
Source: Front Public Health. 2023 Mar 27;11:1108465. doi: 10.3389/fpubh.2023.1108465 (PMC10083433; doi:10.3389/fpubh.2023.1108465)
Supplement: Supplementary file 1 [file Data_Sheet_1.ZIP › 3. Supplementary Tables 1&2.docx]

Supplementary Material

Supplementary Table 1. Baseline characteristics (2016-2018) of study participants (N=2045) by response status during follow-up (2021-2022)

| Characteristics | Total (N=2045) | | Respondents (N=1727) | | Non-respondents* (N=318) | | p-value^##^ |
| --- | --- | --- | --- | --- | --- | --- | --- |
|  | N | % | N | % | N | % |  |
| Age** in years | 44.1 | 13.0 | 44.0 | 12.8 | 44.2 | 14.0 | 0.769 |
| Sex | | | | | | | |
| Men | 929 | 45.4 | 790 | 45.7 | 139 | 43.7 | 0.503 |
| Women | 1116 | 54.6 | 937 | 54.3 | 179 | 56.3 |  |
| Educational status | | | | | | | |
| Up to secondary school | 1514 | 74.0 | 1255 | 72.7 | 259 | 81.5 | 0.001 |
| Graduation and above | 531 | 26.0 | 472 | 27.3 | 59 | 18.5 |  |
| Employment status | | | | | | | |
| Employed | 912 | 44.6 | 774 | 44.8 | 138 | 43.4 | 0.639 |
| Un-employed^ | 1133 | 55.4 | 953 | 55.2 | 180 | 56.6 |  |
| Monthly household income in INR | | | | | | | |
| <=30,000 | 1594 | 77.9 | 1344 | 77.8 | 250 | 78.6 | 0.179 |
| >30,000 | 406 | 19.9 | 349 | 20.2 | 57 | 17.9 |  |
| Consumed Tobacco^^ | 466 | 22.8 | 367 | 21.3 | 99 | 31.1 | <0.001 |
| Consumed Alcohol^^ | 409 | 20.0 | 345 | 20.0 | 64 | 20.1 | 0.951 |
| Presence of at least one comorbidity^#^ | 1821 | 89.1 | 1549 | 89.7 | 272 | 85.5 | 0.029 |
| Presence of gingival/periodontal disease | 1728 | 84.5 | 1451 | 84.0 | 277 | 87.1 | 0.162 |
| * Includes those deceased before January 2020, loss to follow-ups, refusals  **Mean (SD)  ^Unemployed includes homemaker/Student/retired/un-employed  ^^Ever consumed tobacco/alcohol  #Includes hypertension (and pre-hypertension), diabetes (and pre-diabetes), hyperlipidaemia, overweight/obesity, heart disease, kidney disease, stroke, and cancer. All the conditions are based on self-report by the participant following a physician-diagnosis and medication intake except hypertension, diabetes, overweight/obesity, which are also based on measured values. Hypertensive (>=140mm Hg /90mm Hg), pre-hypertensive (SBP: 120 mm Hg to <140 mm Hg or DBP: 80 mm Hg to <90 mm Hg), normotensive (<120 mm Hg/80mmHg). Diabetes (hba1c>=6.5 or Fasting plasma glucose (FPG)>=126mg/dl), Pre-diabetes (hba1c>=5.7 to <6.5) or (FPG 100 mg/dl to <126 mg/dl) and Normoglycemic (hba1c<5.7 and FPG<100 mg/dl). Overweight/obesity BMI >=23kg/m2.  ##p-values for differences in means/median/proportions were from t-test, Wilcoxon Rank test or Chi-Square test respectively  Missing values household income (N=45), BMI (N=11) | | | | | | | |

Supplementary Table 2. Evidence from literature on the association between periodontal disease and COVID-19

| **Author, Year, Settings** | **Study Design** | **Timeline** | **Sample size** | **Confounders assessed** | **Estimates compared to healthy controls**  **(odds ratio (OR - 95% CI)/Incidence Risk ratio (IRR – 95% CI))** |
| --- | --- | --- | --- | --- | --- |
| Alnomay N et al, 2022,  Saudi Arabia (14) | Retrospective cohort | Jan 2020 - July 2021 | N=188 COVID-19 cases | Age, sex, smoking habits, laboratory results, (White Blood Cell count, HbA1c, C-reactive protein (CRP), and Erythrocyte sedimentation rate (ESR)) and comorbid conditions | Periodontal disease (PD) - 52.7%  COVID-19 complications: OR -3.063 (95% CI 1.150-8.157) |
| Marouf et al, 2021, Qatar (19) | Case-Control | Feb-July 2020 | N=568 COVID-19 cases with & without complications  (40 with complications) | Age, sex, BMI, smoking habits, and comorbid conditions | PD – 45.4% (43% in controls)  Mortality: (OR = 8.81, 95% CI 1.00–77.7), ICU admission (OR = 3.54, 95% CI 1.39–9.05) and need for assisted ventilation (OR = 4.57, 95% CI 1.19–17.4) |
| Larvin et al, 2020*, UK (13) | Nested Case-Control | Mar - Jun2020 (2006-2010 baseline recruitment) | N=13253 participants (1616 COVID-19 cases) | Demographics, BMI, biomarkers (blood pressure and resting heart rate), lifestyle factors and comorbid conditions | Painful gums - 2.4%, Bleeding gums - 8.7%, Loose teeth - 2.7%  Loose teeth OR of COVID-19 infection (OR: 1.15, 95% CI 0.84–1.59), hospital admission (OR = 1.55, 95% CI 0.87–2.77), mortality (OR: 1.85; 95% CI 0.92–2.72).  Bleeding/painful gums OR of COVID-19 infection (OR: 1.10, 95% CI 0.72–1.69), hospital admission (OR: 0.90, 95% CI 0.59–1.37), mortality (OR: 1.71, 95% CI 1.05–2.72) |
| Said et al, 2022, Qatar (18) | Case control | Mar 2020 - Feb 2021 | N=1325 COVID-19 cases with & without complications  (71 with complications) | Demographic and comorbid conditions | PD - 45.6%  Non treated PD mechanical ventilation: OR 3.91, 95% CI 1.21-12.57  Treated PD mechanical ventilation: OR 1.28, 95% CI 0.25-6.58 |
| Kamel et al, 2021, Egypt (51) | Cross sectional | Apr – Jul 2020 | N=308 COVID-19 cases | Age, sex, weight, height, education, and general health condition | Poor oral health - 20.8%  Incidence of the delayed recovery period (six weeks) in severe COVID-19 patients 45.0% and 6.1% in mild cases (p <0.001) |
| Gardelis et al, 2022, Geneva (52) | Pilot Cross sectional | Apr 2020 -Jun 2021 | N=30 COVID-19 cases | Comorbid conditions, medication, and smoking habits | PD - 100% cases (27% severe PD) |
| Anand et al, 2022, India (12) | Case-Control | Aug 2020 -Feb 2021 | N=150 participants  (79 COVID-19 cases) | Age, sex, COVID-19 symptoms, comorbid conditions, tobacco consumption, and oral hygiene habits (types and frequency) | Gingivitis - 73.3%, PD - 30.7%.  COVID-19 risk OR mean plaque scores≥1: 7.01, 95% CI 1.83-26.94, gingivitis: 17.65, 95% CI 5.95-52.37, mean CAL≥2mm: 8.46, 95% CI 3.47-20.63, and PD: 11.75, 95% CI 3.89-35.49 |
| Costa et al, 2022, Brazil (15) | Prospective observational study | Aug 2020 -Mar 2021 | N=128 COVID-19 cases | Age, sex, race, education, monthly family income, BMI, smoking habits, alcohol intake, comorbid conditions, pregnancy, and sexually transmitted diseases | Gingivitis - 43.8%, PD - 47.9%  ICU admission IRR 1.44, 95%CI 1.07–1.95, critical symptoms 2.56, 95%CI 1.44–4.55, risk of death 2.05, 95%CI 1.12‐3.76 |
| Gupta S et al, 2022, India (17) | Cross sectional | Jan – Feb 2021 | N=82 COVID-19 cases | Age, sex, smoking habits, and comorbid conditions | Gingivitis - 25.6%, PD - 26.9%  Bleeding on probing OR of assisted ventilation: 4.14 (95% CI 1.51–11.34), hospital admission: 3.18 (95% CI 1.24–8.15), COVID-19 pneumonia: 3.63 (95% CI 1.18–11.12) Gingival recession OR of assisted ventilation: 8.22 (95% CI 2.55–26.45), COVID-19 pneumonia: 6.50 (95% CI 2.12–19.90)  >4 missing teeth OR of hospital admission: 12.52 (95% CI 1.48–105.58).  PD OR of assisted ventilation: 7.45 (95% CI 2.71–20.45), hospital admission: 36.52 (95% CI 4.62–288.64), death: 14.58 (95% CI 1.69–125.33) and COVID-19 pneumonia: 4.42 (95% CI 1.57–12.45). |
| Kaur A et al, 2022, India (53) | Cross sectional | Mar 2021 | N=116 COVID-19 cases | Age, gender, smoking habits, BMI, and comorbid conditions | PD in moderate COVID-19 group - 81%, mild group - 46.2%  OR of severe PD were 6.32 times more in subjects belonging to moderate Vs mild group. |
| Mishra S et al, 2022, India (16) | Cross sectional | Apr - Aug2021 | N=294 COVID-19 cases | Age, sex, BMI, smoking habits, comorbid conditions, and pregnancy | PD - 50.68% (87.5% in severe group)  OR of having severe COVID-19: 2.8133 (95% CI 0.4077–19.7523) |
| Katz et al,2022, Florida (54) | Cross sectional | NA | N=889 COVID-19 cases | Age, race, sex, smoking habits, and comorbid conditions, and dental caries | PD - 0.77%  Unadjusted OR of COVID risk 1.1 (95% CI 0.3, 4.2). OR for COVID-19 risk (adjusted for smoking): 4.7 (2.32-9.4) |
| Wang et al, 2021, COVID-19 Host Genetics Initiative (55) | Mendelian Randomisation | NA | N=1299010 for COVID-19 susceptibility  N=908494 for COVID-19 severity  N=975 for PD assessment | | Inverse Variance Weighted (IVW) OR for COVID-19 susceptibility 1.024 (95% CI 1.004–1.045), weighted median method OR1.029 (95% CI 1.003–1.055).  IVW OR for COVID-19 severity 1.025 (95% CI 1.001–1.049), weighted median OR 1.030 (95% CI 1.003–1.058) |
| *The only population-based study from UK Biobank, all others are hospital based | | | | | |
