# Supplementary figures and images for: Epidemiological pattern of COVID-19 and its association with periodontal health in an urban Indian cohort
Source: Front Public Health. 2023 Mar 27;11:1108465. doi: 10.3389/fpubh.2023.1108465 (PMC10083433; doi:10.3389/fpubh.2023.1108465)

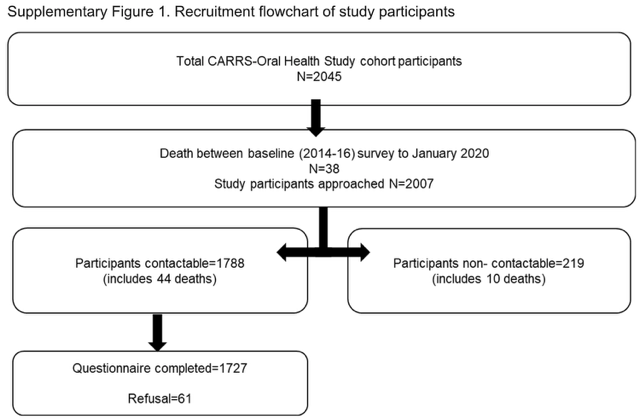

Supplement: Supplementary file 1 [file Data_Sheet_1.ZIP › 4. Suppl Figure 1.tiff]

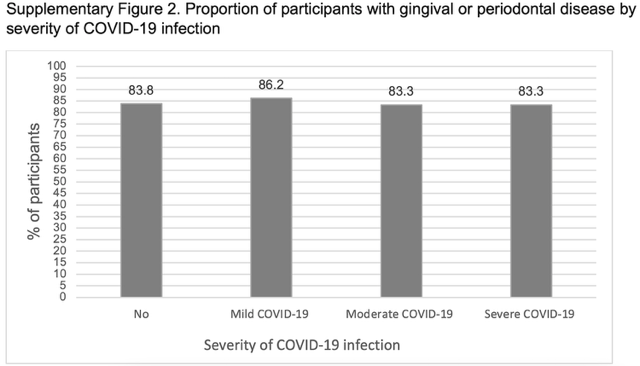

Supplement: Supplementary file 1 [file Data_Sheet_1.ZIP › 5. Suppl Figure 2.tiff]
